# Detection of plasma Aβ seeding activity by a newly developed analyzer for diagnosis of Alzheimer’s disease

**DOI:** 10.1186/s13195-022-00964-2

**Published:** 2022-02-02

**Authors:** Jianping Jia, Tingting Li, Jianwei Yang, Baian Chen, Wei Qin, Cuibai Wei, Yang Song, Qigeng Wang, Yan Li, Longfei Jia

**Affiliations:** 1 Innovation Center for Neurological Disorders and Department of Neurology, Xuanwu Hospital, Capital Medical University, National Clinical Research Center for Geriatric Diseases, Beijing, China; 2 Beijing Key Laboratory of Geriatric Cognitive Disorders, Beijing, China; 3 Clinical Center for Neurodegenerative Disease and Memory Impairment, Capital Medical University, Beijing, China; 4 Center of Alzheimer’s Disease, Beijing Institute of Brain Disorders, Collaborative Innovation Center for Brain Disorders, Capital Medical University, Beijing, China; 5 Department of Neurobiology, Beijing Key Laboratory of Neural Regeneration and Repair, School of Basic Medical Sciences, Capital Medical University, Beijing, China

**Keywords:** Alzheimer’s disease, Amyloid-β, Seeding activity, Biomarker, Blood

## Abstract

**Objective:**

To evaluate the diagnostic value of plasma β-amyloid (Aβ) seeding activity measured using a newly developed instrument to distinguish Alzheimer’s disease (AD) from other forms of dementia.

**Methods:**

Seventy-nine AD patients, 64 non-AD dementia (NADD) patients, and 75 cognitively normal (NC) subjects were recruited in the study. To measure the levels of Aβ seeding activity in the plasma samples, we have developed an AD-seeds protein analyzer. We used receiver operating characteristic (ROC) curves to quantify the ability of plasma Aβ seeding activity to distinguish between AD and NADD or NC individuals. Spearman’s correlation was used to examine the associations between plasma Aβ seeding activity and global cognitive function or conventional AD biomarkers.

**Results:**

The Aβ seeding activities were 0.83 (0.58–1.16) A.U. in AD, 0.42 (0.04–0.74) A.U. in NADD and 0.42 (0.09–0.69) A.U. in NC, respectively. The Aβ seeding activity was able to identify AD patients and distinguish them from NC or NADD with high accuracy (AUC = 0.85–0.86). In addition, the plasma Aβ seeding activity showed a strong correlation with cognitive performance (mini-mental state examination, *r* = − 0.188; Montreal cognitive assessment, *r* = − 0.189; clinical dementia rating, *r* = 0.205) and conventional biomarkers (cerebrospinal fluid [CSF] Aβ42/40, r = -0.227; CSF T-tau/Aβ42, *r* = 0.239; CSF P-tau/Aβ42, *r* = 0.259).

**Conclusion:**

Our results confirmed that plasma Aβ seeding activity is an antibody-free and low-cost biomarker for the diagnosis of AD.

**Trial registration:**

Trial registration number NCT04850053

## Introduction

The number of studies investigating the development of biomarkers for the early diagnosis of Alzheimer’s disease (AD) has increased throughout medical communities worldwide [1]. Among the novel approaches employed are those exploiting the polymerization property of β-amyloid (Aβ), that is, the ability to act as seeds that can recruit other soluble monomers and assemble to form aggregates [2]. Aβ seeds, including small, soluble aggregates, and large, insoluble fibrils, are the major toxic substances associated with the pathology of AD [3, 4]. Extensive evidence suggests that soluble Aβ seeds circulate in biological fluids such as the cerebrospinal fluid (CSF) [5] and blood [6, 7]. Thus, Aβ seeds have become a promising candidate biomarker for the specific biochemical diagnosis of AD. In this context, one important objective in current research is to detect and quantify small amounts of Aβ seeds present in the biological fluids of AD patients for clinical applications.

Efforts to measure Aβ seeds in humoral fluids are currently underway. Recently, a novel assay has been developed to detect minute amounts of Aβ seeds in the CSF using protein misfolding cyclic amplification (PMCA) technology with high sensitivity and specificity [5]. However, because of the invasive nature of lumbar puncture of CSF collection, this method is not practical to collect the CSF for routine early detection of Aβ seeds during clinical visits or for serial evaluation during clinical trials. Given that blood sampling is relatively easier and much less invasive, blood Aβ seeds may serve as a more practical diagnostic biomarker for AD. Thus, a clinically applicable method to measure Aβ seeding activity in the blood is required to properly diagnose and monitor AD. In this context, we have developed an AD-seeds protein analyzer, in which a fluorescence microplate reader was combined with an oscillating mixer or water-bath-type ultrasonicator. In AD-seeds protein analyzer, seeding activities in the blood samples from patients can be amplified by several orders of magnitude in vitro using either sonication or shaking to accelerate polymerization. If the same amount of monomeric Aβ were spiked into AD and non-AD plasma samples, a different kinetic pattern of aggregation would be observed between the two groups. The AD-seeds protein analyzer measures Aβ seeds in the blood, unlike conventional techniques, such as single-molecule array (Simoa) technology [8] or mass spectrometry-based assays [9], which directly measure Aβ molecules. In the case of analytic techniques based on the immunoassay platform, the requirement of expensive equipment limits their widespread application. In contrast, our AD-seeds protein analyzer is an antibody-free and cost-effective approach for measuring blood biomarkers.

In this study, we aimed to evaluate the analytic performance of AD-seeds protein analyzer to measure Aβ seeding activity in the plasma of AD patients and show its performance in the differential diagnosis of the disease. We distinguished AD patients from non-AD individuals by measuring the Aβ seeding activity in plasma samples after spiking synthetic Aβ. Our hypothesis was that the plasma Aβ seeding activity from AD patients would be different from that in non-AD subjects.

## Materials and methods

### Biological samples

In the present study, we used plasma samples from 79 patients with the diagnosis of probable AD as defined by the National Institute on Aging-Alzheimer’s Association guidelines [10]. The non-AD individuals included 75 subjects with cognitively normal (NC) and 64 with non-AD dementia (NADD) (i.e., vascular dementia [VaD], frontotemporal dementia [FTD], dementia with Lewy bodies [DLB], and other dementia types). Table 1 displays a summary of the demographic characteristics of these subjects. Subjects were recruited consecutively from the inpatient and outpatient departments of the Xuanwu Hospital, Capital Medical University between January 2018 and December 2020. All subjects underwent neuropsychological assessments including the mini-mental state examination (MMSE) and the Montreal cognitive assessment (MoCA) for global cognitive functions, and clinical dementia rating (CDR) scale for clinical disease severity. Most (94.5%) participants were assessed using apolipoprotein E (*APOE*) genotyping. Conventional biomarkers of AD (Aβ42, P-tau, and T-Tau) were analyzed if the CSF samples were available. This study was approved by the Medical Ethics Committee of Xuanwu Hospital, and written informed consent was obtained from every participant. The investigators were blinded to sample source information during the experiments and analysis.

**Table 1:** Baseline characteristics of study population

Characteristic	AD (*n*=79)	NADD (*n*=64)	NC (*n*=75)	*p*
Man, *n* (%)	34 (43.0)	39 (60.9)	10 (13.3)	<0.001
Age, years	64.15±8.75	63.16±7.64	61.85±5.47	0.108
Education, years	11.11±4.07	9.15±4.64	13.48±2.91	<0.001
Disease duration, years	2.76±1.51	2.28±1.90	NA	<0.001
*APOE* ε4, *n* (%)	38 (55.1)	12 (19.4)	14 (18.7)	<0.001
MMSE	17.99±7.38	16.66±8.07	29.76±0.46	<0.001
MoCA	13.76±6.96	11.92±6.71	28.52±1.16	<0.001
CDR	1.42±0.92	1.49±0.97	0	<0.001
Aβ42, pg/ml	341.74±152.1	514.32±246.80	NA	<0.001
P-tau181, pg/ml	163.98±84.62	69.83±64.43	NA	<0.001

*Abbreviations*: *AD*, Alzheimer’s disease; *APOE*, apolipoprotein E; *CDR*, clinical dementia rating; *MMSE*, Mini-Mental State Examination; *MoCA*, Montreal cognitive assessment; *NADD*, non-AD dementia; *NA*, not applicable; *NC*, normal cognition

### Processing of plasma samples

**Fig. 1:**
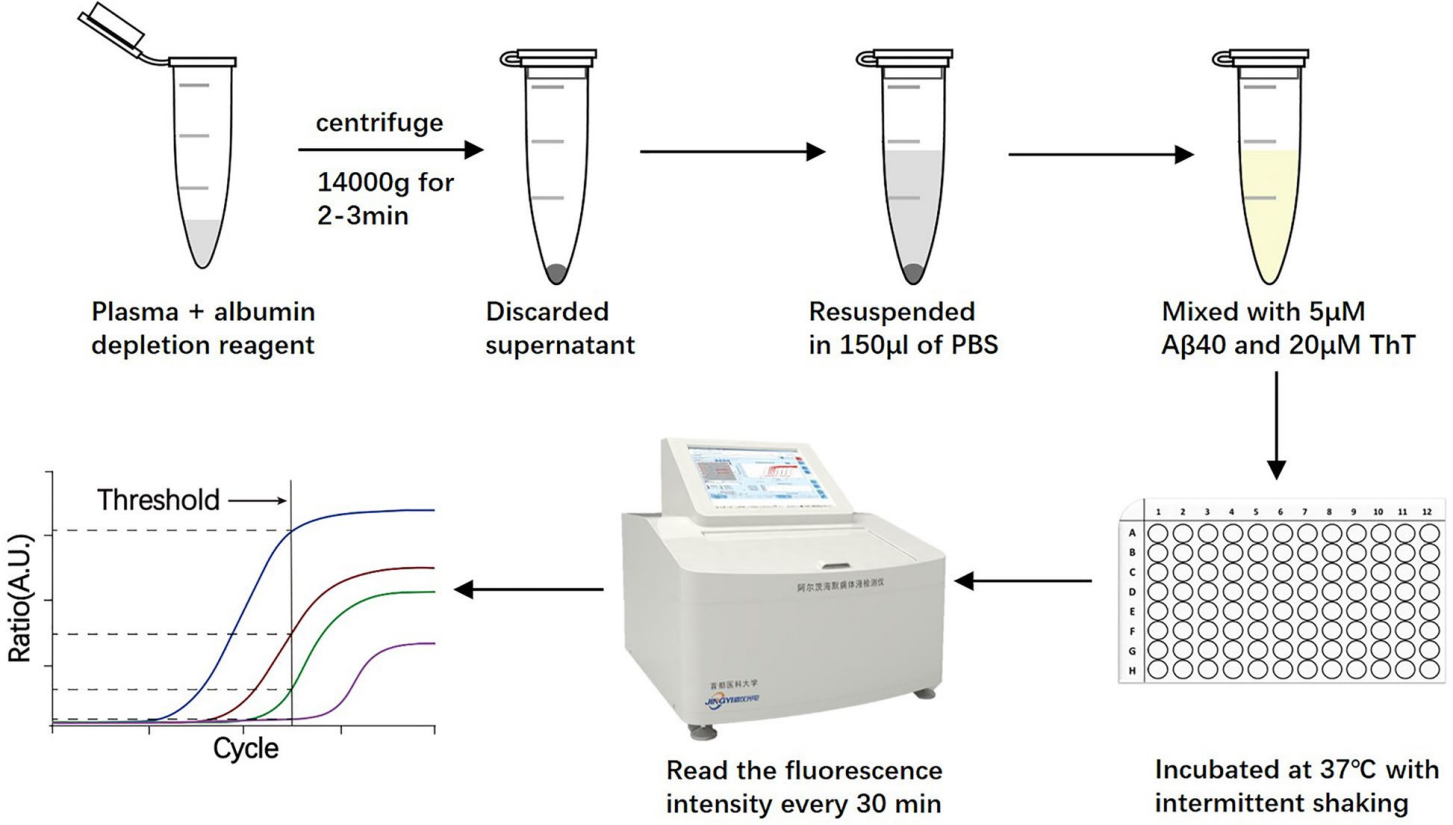
Schematic representation of steps during processing of plasma samples and the AD-seeds protein analyzer. To remove albumin, samples of plasma were incubated with 1 volume of albumin depletion reagent. After centrifugation, the pellet was resuspended directly in PBS, and then mixed with 5 μM Aβ40 and  20 μM ThT. Samples were subjected to a round of 130 cycles. The kinetics of fibril formation was monitored real time by the reading of the fluorescence intensity every 30 min using 440±10 nm excitation and 480±10 nm emission wavelengths. Aβ, β-amyloid; AD, Alzheimer’s disease; PBS, phosphate buffered saline

Plasma samples were processed to remove albumin that interfered with the Aβ aggregation [11, 12] (Fig. 1). Briefly, 20 μL of a sample was mixed with 1 volume of albumin depletion reagent (Invent, USA) by pipetting the solution up and down for 10–20 times. Thereafter, samples were centrifuged at 14,000*g* for 2–3 min, the supernatant was discarded, and the pellet was resuspended in 150 μL of phosphate buffered saline (PBS).

### Plasma Aβ seeding activity measurements

AD-seeds protein analyzer is based on the seeding-nucleation mechanism to cyclically amplify the process of protein misfolding and aggregation, enabling efficient amplification of small quantities of Aβ seeds [6]. Therefore, we can detect the presence of Aβ seeds in peripheral blood by measuring the seeding activity in a plasma sample over a monomeric Aβ substrate. Briefly, samples of seed-free, monomeric Aβ40 (Abcam, UK) at a concentration of 5 μM in PBS buffer, pH 7.4 were placed in opaque 96-well plates in the presence of 20 μM Thioflavin-T (ThT) at a final volume of 200 μL. For each test, we added 20 μL of plasma samples from patients and control subjects. Each sample was run in triplicate. Samples were subjected to cyclic agitation (1 min at 500 rpm followed by 29 min without shaking) at 37 °C. The increase of ThT fluorescence was monitored every 30 min (440 ± 10 nm excitation, 480 ± 10 nm emission).

### Determination of the kinetic parameter

The differences in the Aβ seeding activity between different samples were evaluated via P42 (We have selected the fluorescence amplitude ratio throughout all cycles for analysis. Because P42 is the best point to distinguish the AD, NADD and NC, we determine it as a threshold.) estimation. P42 corresponds to the extent of aggregation (measured as ThT fluorescence) at 42 h. Importantly, we divided the ThT fluorescence in plasma samples by that in blank samples to calculate a standard ratio, so as to minimize intra-individual variations.

### CSF analysis

CSF samples were collected in 10 mL polypropylene tubes and transported to the laboratory within 2 h. Samples were then centrifuged for 10 min at 2000*g* at 4°C. All but the bottom 500 μL was aliquoted (500 μL) into 1.5ml polypropylene tubes and immediately stored at – 80 °C until analysis. CSF T-tau, P-tau, Aβ42, and Aβ40 peptide concentrations were measured by the human Luminex 4-plex xMAP assay (Millipore; US) according to the manufacturer’s instructions. CSF analyses were performed blinded to the clinical diagnoses.

### Statistical analysis

Group-wise comparisons of demographic, clinical, and biomarker characteristics were assessed using Mann–Whitney *U* tests for continuous and chi-squared test for categorical variables. Receiver operating characteristic (ROC) curves and binary logistic regression (with age, sex, education, and *APOE ε4* status as covariates) were applied to analyze the diagnostic value of plasma Aβ seeding activity in differentiating AD samples from NC or NADD. In individuals who had cognitive test scores and CSF assessments, Spearman’s correlation analysis was performed to examine the associations between plasma Aβ seeding activity and global cognitive function or conventional AD biomarkers. The level of significance was set at *p* < 0.05. All statistical analyses were performed using the GraphPad Prism 7.0 and the IBM SPSS Statistics 25.0.

### Data availability

All data generated and/or analyzed during this study are included in the article. Any additional information required are available from the corresponding author on reasonable request.

## Results

### Demographic characteristics of the included participants

The between-group difference in age was not significant. However, there were significant differences in sex, education, and disease duration among groups. The number of *APOE* ε4 carriers (homozygote or heterozygote) was higher in the AD patients (55.1%) than in the NC (18.7%) or NADD individuals (19.4%). As expected, the AD and NADD patients exhibited lower performance on the global cognitive function test than the control group did. Demographic, clinical, and cognitive characteristics of the research participants are summarized in Table 1.

### Detection of Aβ seeding activity in the plasma of AD patients

**Fig. 2:**
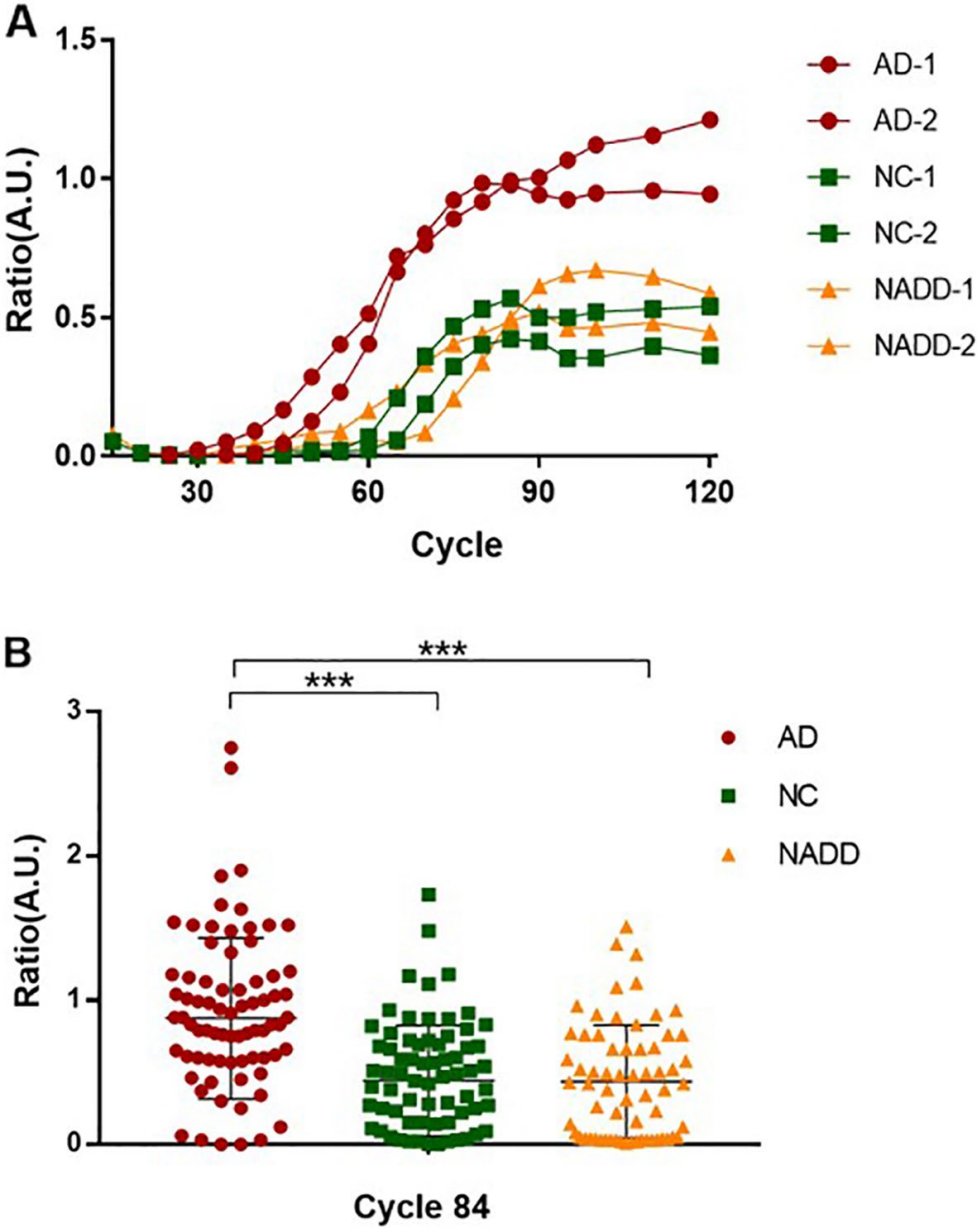
Results of AD, NC, and NADD patients. Representative aggregation curves of seed-free Aβ40 in the presence of plasma samples from AD patients, NC individuals, and NADD patients (**A**). The values represent the average and SE of six different patients, representative of the average results in each group. The extent of amyloid formation obtained after 84 cycles, i.e., 42 h of incubation (P42), is measured in each patient (**B**). AD, Alzheimer’s disease; A.U., arbitrary units; NADD, non-AD dementia; NC, cognitively normal. ***p<0.001

Figure 2A shows the average kinetics of aggregation in six representative samples from the AD patients, NC, and NADD patients. The result indicates that plasma from AD patients significantly accelerates Aβ aggregation as compared to those from NC and NADD patients (*p* < 0.001).

### Detection of Aβ seeding activity toward an accurate diagnosis of AD

**Fig. 3:**
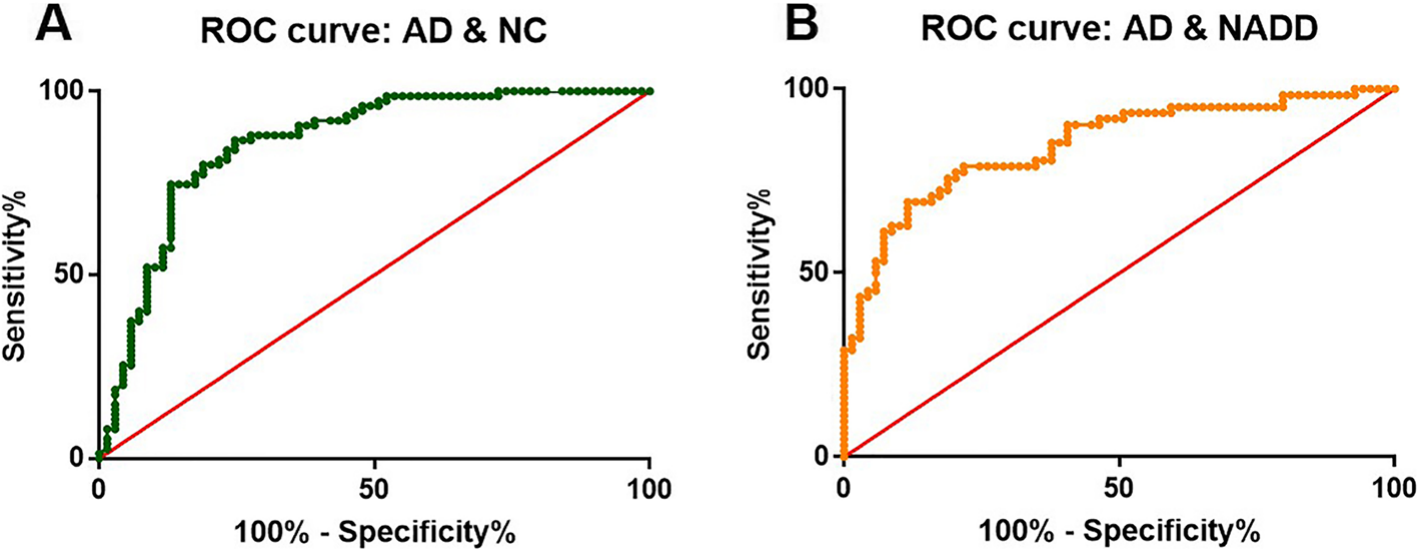
ROC curve analysis of sensitivity and specificity of the composite score of combing Aβ seeding activity and *APOE* ε4 status. To evaluate the performance of the composite score to distinguish AD patients from non-AD individuals, we plotted the true positive rate (sensitivity) in function of the false positive rate (specificity) for different cut-off points. The performance of the test, estimated as the area under the curve was 0.86 (95% confidence interval [CI]: 0.80–0.92; sensitivity: 81.2%, specificity: 80.0%, positive predictive value: 78.9%, negative predictive value:82.2%) and 0.85 (95% CI: 0.78–0.92; sensitivity: 81.2%, specificity: 75.8%, positive predictive value: 78.9%, negative predictive value:78.3%) when AD compared with NC or NADD, respectively. AD, Alzheimer’s disease; Aβ, β-amyloid; ROC, receiver operating characteristic; NADD, non-AD dementia; NC, cognitively normal

To determine the effect of individual samples on Aβ aggregation, we estimated the P42, defined as the extent of Aβ aggregation at 42 h (Fig. 2B). By comparing the P42 parameter among the groups, a highly significant difference was observed between AD (0.83 [0.58–1.16] A.U.]) and non-AD samples from NC individuals (0.42 [0.09–0.69] A.U.]), or NADD patients (0.42 [0.04–0.74] A.U.). Using the P42 values, we calculated the diagnostic performance of the plasma Aβ seeding activity. To determine the performance of the test, we carried out a detailed statistical analysis using a ROC analysis (Fig. 3). We estimated an area under the curve (AUC) value of 0.86 (95% confidence interval [CI]: 0.80–0.92) in relation to the age-matched NC, whereas 0.85 (95% CI: 0.78–0.92) to differentiate for AD patients from NADD patients. If confirmed with a larger number of patients, the ability of Aβ seeding activity to distinguish AD from non-AD individuals can have important clinical application.

### Correlation of plasma Aβ seeding activity and global cognitive function or conventional AD biomarkers

All participants underwent clinical evaluations using global cognitive function tests. Thus, no samples were excluded from this analysis. The plasma Aβ seeding activity values were strongly correlated with the scores on the MMSE (*r* = − 0.188, *p* = 0.005), MoCA (*r* = − 0.189, *p* = 0.005), and CDR (*r* = 0.205, *p* = 0.002) in the whole group (Table 2).

**Table 2:** Association of plasma Aβ seeding activity values with global cognitive function and CSF core biomarkers

	MMSE	MoCA	CDR	CSF Aβ42/40	CSF T-tau/Aβ42	CSF P-tau/Aβ42
Seeding activity	− 0.188 (0.005)	− 0.189 (0.005)	0.205 (0.002)	− 0.227 (0.013)	0.239 (0.008)	0.259 (0.004)

*Abbreviations*: *CDR*, clinical dementia rating; *CSF*, cerebrospinal fluid; *MMSE*, Mini-Mental State Examination; *MoCA*, Montreal cognitive assessment

Subjects without CSF data were excluded from the correlation analysis between plasma Aβ seeding activity and conventional AD biomarkers. Thus, 110 subjects (AD, n = 56; NADD, n = 64) were included in the correlation analyses. The plasma Aβ seeding activity values were strongly correlated with CSF Aβ42/40 (*r* = − 0.227, *p* = 0.013), CSF T-tau/Aβ42 (*r* = 0.239, *p* = 0.008), and CSF P-tau/Aβ42 (*r* = 0.259, *p* = 0.004) (Table 2).

## Discussion

In this study, Aβ seeding activities in plasma of AD and non-AD individuals were measured using the novel AD-seeds protein analyzer. We demonstrated that the plasma Aβ seeding activity had a robust performance in distinguishing AD patients from non-AD individuals. The current observations supported the notion that plasma Aβ seeding activity could serve as a potential blood-based biomarker for the sensitive diagnosis of AD.

Previous studies have shown that injecting AD brain extracts in the brains in mouse models of AD could accelerate amyloid deposition through prion-like mechanisms [13–16]. Recently, several reports have shown that infusion of blood from mice displaying cerebral amyloidosis accelerated amyloid pathology in animal models of AD, supporting the concept that Aβ seeds are present in the blood and are implicated in the development of AD [17]. Another report has showed that wild-type mice could develop brain amyloidosis after parabiosis in APP/PSEN1 transgenic mice, further supporting the important role of circulating Aβ on brain pathology [18]. In this study, we developed a new instrument to measure Aβ seeding capability in the plasma of AD patients. We found that plasma Aβ seeding activity demonstrated high diagnostic accuracy in detecting AD in the dementia stage of the disease, which makes plasma Aβ seeding activity a potential candidate for an AD-specific blood biomarker. More importantly, plasma Aβ seeding activity showed high diagnostic accuracy in distinguishing between AD and NADD patients. The capability of plasma Aβ seeding activity to distinguish AD patients from NADD patients might be valuable in clinical practice and trials. Note that the differential diagnosis of AD among other dementias is difficult using clinical testing [19]. Plasma Aβ seeding activity may be used to improve the accuracy of differential diagnosis of dementia patients. Together, our findings provide the proof-of-principle basis for the detection of blood-based Aβ seeds for AD diagnosis.

Owing to its low concentrations in the blood, detecting crude Aβ seeds in plasma has been a challenge, especially in the presence of several interfering factors, such as albumin and immunoglobulin, at high concentrations [20, 21]. Given that albumin is the most abundant plasma protein reported to bind Aβ impeding its aggregation [11, 12], we determined the sensitivity of our assay in the absence of albumin. Even at ultralow concentrations, the formation of Aβ aggregates in the blood of AD patients may be initiated via incubation with spiked synthetic Aβ peptides [21]. In previous studies, researchers were able to differentiate Aβ oligomerization tendency by spiking Aβ42 into the plasma of AD patients and control individuals [20–24]. The Aβ oligomerization differences in plasma have significant potential in AD diagnosis. In our study, Aβ seeding activity levels in the plasma were increased in the AD patients, in agreement with previous reports mentioned before.

It is also necessary to identify markers that correlate with the severity of dementia in AD patients. In this study, the correlation coefficient indicated a moderately strong relationship between the elevated plasma Aβ seeding activity and the decreased general cognitive level. Previous studies have demonstrated that the plasma Aβ oligomerization tendency was correlated with the general cognitive function and episodic memory [23]. Similarly, measuring the plasma Aβ seeding activity using our instrument could be associated with symptom severity, which requires further investigation for its potential use in monitoring disease progression or as a prognostic biomarker of AD. Furthermore, our findings show a positive correlation between plasma Aβ seeding activity and the established CSF biomarkers, suggesting that our instrument may offer a good opportunity for a much-needed sensitive biochemical diagnosis of AD. This is consistent with previous studies that found the levels of Aβ oligomerization in the plasma were correlated strongly with CSF core biomarkers [20]. Moreover, studies conducted in animal models showed that decreasing Aβ seeding activity in plasma was significantly correlated with the reduction of brain Aβ deposits [6]. Overall, these findings indicate that plasma Aβ seeding activity mirror AD pathology and have clinical potential for AD diagnosis.

### Limitations

Although the present study revealed the interesting potential of plasma Aβ seeding activity to serve as a blood-based biomarker, our findings have limitations and should be interpreted with caution. For instance, compared with AD and NC, the number of cases with NADD, such as VaD, FTD, and DLB, is relatively small. Our findings need to be further validated with a larger cohort and in longitudinal studies. In addition, individuals with preclinical AD could not be included. Further studies are needed to determine if plasma Aβ seeding activity can be detected in blood preclinically in AD. Another limitation of our study is that the characteristics of samples used in the analysis were mainly based on the clinical diagnosis. Thus, a follow-up or pathologically confirmed diagnosis of each case will be critical to validate the differential diagnostic utility of the plasma Aβ seeding activity. It is possible that the diagnostic accuracy of the test could be even higher if neuropathologically confirmed samples were used.

## Conclusions

Plasma samples of AD and non-AD subjects were differentiated using the AD-seeds protein analyzer, which measured the Aβ seeding activity in the plasma. Furthermore, plasma Aβ seeding activity was found to have a robust performance in the differential diagnosis of AD from non-AD individuals. Based on the current findings, measuring the Aβ seeding activity in plasma could be a simple and reliable blood-based diagnostic biomarker for AD. However, further studies are required to elucidate the mechanisms underlying plasma Aβ seeding activity. Longitudinal studies undertaken during the predementia stage of AD should also be conducted to assess clinical applications of this biomarker for early detection and monitoring of this disease.

## Data Availability

The datasets used during the current study are available from the corresponding author on reasonable request.

## Abbreviations

AD: Alzheimer’s disease
Aβ: β-amyloid
NADD: Non-AD dementia
NC: Cognitively normal
ROC: Receiver operating characteristic
CSF: Cerebrospinal fluid
PMCA: Protein misfolding cyclic amplification
VaD: Vascular dementia
FTD: Frontotemporal dementia
DLB: Dementia with Lewy bodies
MMSE: Mini-Mental State Examination
MoCA: Montreal cognitive assessment
CDR: Clinical dementia rating
APOE: Apolipoprotein E
PBS: Phosphate buffered saline
ThT: Thioflavin-T
AUC: Area under the curve
